# Biomechanical variables in Icelandic horse riders and the effect on tölt performance: A pilot study

**DOI:** 10.1371/journal.pone.0287748

**Published:** 2023-06-27

**Authors:** J. K. Sätter, K. McGawley, M. Connysson, C. A. Staunton

**Affiliations:** 1 Swedish Winter Sports Research Centre, Department of Health Sciences, Mid Sweden University, Östersund, Sweden; 2 Department of Anatomy, Physiology and biochemistry, Swedish University of Agricultural Sciences, Wången, Alsen, Sweden; Ningbo University, CHINA

## Abstract

**Aim:**

To identify how riding rein direction (left and right) and rider asymmetry affect tölt performance in Icelandic horses.

**Methods:**

Two horses were ridden in tölt by four riders on both left and right reins. Riders wore pressure insoles that measured the total absolute force (F_Abs_) and absolute force difference (F_Diff_) in their left and right feet in the stirrups. A 3D motion-analysis system recorded the degrees of side-to-side movement in the pelvis (RollP) and in the thoracolumbar region (RollT). Lateral advanced placement (LAP) and duty factor (DF) were calculated to determine tölt performance. One-way ANOVAs were used to assess the effect of rein direction on rider asymmetry variables (F_Abs_, F_Diff_, RollP and RollT) and tölt performance (LAP, DF) on a group level (n = 8). Within-subject Spearman rank correlations (ρ) were computed to determine the effect of rider asymmetry variables on tölt performance on an individual level.

**Results:**

LAP was closer to 25% on the left rein compared to the right rein (mean difference: 1.8±1.2%; F_(1,7)_ = 16.333; p = 0.005, η^2^_p_ = 0.700). In addition, DF was lower on the left rein compared to the right rein (mean difference: 1.9±0.8%; F_(1,7)_ = 41.299; p<0.001, η^2^_p_ = 0.855). Individual relationships between RollT and LAP ranged from small negative to very large positive and reached significance for one rider (*ρ =* 0.730; p = 0.040). Individual relationships between RollP and DF ranged from very large negative to very large positive and reached significance for two riders (*ρ =* 0.731; p = 0.040; *ρ = -*0.723 p = 0.043).

**Conclusion:**

Rein direction might influence tölt performance. Individual relationships between rider asymmetry and tölt performance were highly variable and reached significance in some instances, indicating that the relationship between rider asymmetry and tölt performance is highly individual. This type of biomechanical data can be used to provide valuable feedback to guide equestrians and coaches.

## Introduction

Equestrian riders use their whole body to communicate with horses in mounted disciplines such as dressage, jumping or Icelandic horse riding, where a horse and a rider should move together in harmony [1]. A rider uses various methods to communicate with a horse, by distributing the body weight in the saddle [2], by applying pressure with the legs on a horse’s side [3] or by using the reins to interact with a horse’s mouth [4, 5]. To apply these methods correctly, riders must be aware of horse behaviour and learning theory [6]. The most common learning theory during riding is negative reinforcement, which is the release of pressure (the cue) when a horse performs the desired task [7]. To achieve harmony, the release of pressure with the correct timing and placement is necessary. A novice rider responds to the movements of a horse and typically lags behind a horse’s movements, whereas an expert rider can follow and communicate with the horse with correct timing [1]. A horse can adopt different movement patterns with different riders, showing that the rider influences how the horse moves [8, 9].

A rider’s movements should be controlled when communicating with a horse and symmetry is important, since an asymmetric or one-sided rider who is unable to perform equivalent actions on the left and right sides of their body may affect the horse negatively. For example, uneven stirrup length or uneven pressure under the saddle between the left and right sides can cause movement asymmetries in horses when trotting [11]. This unevenness might cause asymmetries in the range of motion of the thoracolumbar-spine and affect fetlock extension in the hind or front legs [10, 11]. Furthermore, riders leaning to one side can create higher forces in the stirrups [12], which might unbalance the horse [11]. Researchers have suggested that a rider’s straightness/crookedness potentially has a short-term influence on performance and a long-term effect on the musculoskeletal health of both the horse and the rider [13, 14].

A rider should be able to control pelvic movements in all the three dimensions, i.e., elevation and depression of left and right sides (roll), left and right rotation, and posterior and anterior tilt), equally on both sides [15]. This is a key factor not only to be able to follow the horse’s movements but also to guide the horse to perform the gaits correctly, to turn on a circle and to collect [16]. Despite this, research has indicted that few riders can control pelvic movements in three dimensions [17].

Icelandic horses have five gaits: walk, trot, canter, tölt and pace. Tölt is defined as a running walk or an ambling gait, with suspension at the front or back (called ‘half- suspension’), and a consistent rhythm of footfall of right hind, right front, left hind, left front [18]. The definition of true tölt stipulates a presence of both ipsilateral and diagonal stance phases and no full suspension of all four feet at the same time [19]. In competition, true tölt must be demonstrated to score the highest points [20]. For the highest score, tölt should be of a regular rhythm so that the temporal separation between the initiation of the stance phase of each limb is the same. However, the rhythm of footfall (beat) during tölt can be inconsistent. If the diagonal stance phase is prolonged the beat is considered to be closer to trot, whereas if the ipsilateral stance phase is prolonged the beat is considered to be closer to pace [18]. Both of these inconsistencies will receive lower competition scores [20].

Parameters related to the beat and quality of tölt can be calculated [21]. Lateral advanced placement (LAP) is the time passing between ground contacts of the lateral limbs and reflects the proportion of stride duration. In pace, LAP is practically 0%, since the lateral legs hit the ground almost simultaneously, but for pure beat trot the LAP is 50%. In pure tölt and walk, which are four-beat gaits, the LAP should be 25% [22]. A LAP less than 22% is considered ‘pacey’ (i.e., with more lateral movement, until reaching full pace) and more than 28% is considered ‘trott-like’ (i.e., with more diagonal movement, until reaching full trot) [23]. Duty factor (DF) is the percentage of the total stride cycle when a given limb is on the ground, and this can partly define the quality of the tölt. However, there is no defined threshold for good or bad tölt performance. An industry accepted standard of DF > 50% in a single limb suggests that the gait is more of a running walk with tri-pedal support, rather than tölt [22– 25]. A lower percentage means that the horse’s feet are more in the air than on the ground, which is desirable.

Despite research demonstrating how rider asymmetry affects a horse during trotting [10, 11], it remains unknown whether rider asymmetry affects tölt performance in Icelandic horses. Investigating the influence of riders’ movements on tölt performance could provide valuable information to guide equestrians and coaches to improve competition performance. In competition, an Icelandic horse is ridden in both left and right rein directions around an oval-shaped track. Therefore, it is of interest to measure a rider’s biomechanical variation in side-to-side movements (asymmetry) during each riding rein direction to determine the impact on tölt performance. Accordingly, this study aimed to assess the effect of rider asymmetries on tölt performance in Icelandic horses. A secondary aim was to investigate the influence of riding direction (left and right reins) on rider asymmetry and tölt performance.

## Methods

### Study design

This pilot study used biomechanical measurements to examine the effect of rider asymmetry on tölt performance in Icelandic horses. The testing took place in an indoor riding hall approximately 35 × 70 m with a medium-soft fine gravel base, on an oval track simulating a smaller version of an oval competition track [20]. The two straights were approximately 40 m in length and the two curves at each end were approximately 30 m long. Riders were instructed to perform to the best of their ability, as if competing in the section “Any Speed” at a competition, giving them the opportunity to show the horse’s best tölt.

### Participants

Four female Icelandic horse riders (mean ± SD; age: 44 ± 6 years; mass (including tack): 84 ± 5 kg) volunteered to participate in this study. All riders were experienced and had previously competed at national-level events. All riders provided written informed consent before testing. Two Icelandic horses with competition experience (one 11-year-old gelding and one 14-year-old stallion), considered by 4 experienced riding teachers to be capable of performing a clear beat in tölt with a variety of different riders, participated with permission from the owners. The horses were shod with normal steel shoes and were examined by the same veterinarian before each test in the same manner as would occur before competition. This involved visual observation of the horse performing trot in hand on a hard surface. On all occasions, the state of fitness of all horses was considered to be equivalent to that required for competition. In addition, one experienced sport judge was present at all testing sessions and assessed the horses as sound. No objective lameness measurements were made on the horses. The Umeå local ethics committee approved this study, and it was performed in compliance with European Union directives on animal experiments (2010/63/EU; European Union, 2010) and the laws (Swedish Constitution, 1988:534) and regulations (Swedish Board of Agriculture Constitution, 2012:26) governing experiments on live animals in Sweden.

### Procedures

Riders were instructed to warm up and prepare themselves and the horses for approximately 10 minutes prior to testing, as if they were preparing for a competition. All riders brought a saddle of their own choice that was specific for riding Icelandic horses (Hrimnir, Mosfellsbaer, Iceland; Amerigo, Herisau, Switzerland; Equipe, Valdagno, Italy). All riders subjectively placed their saddles in balance [26]. Riders adjusted their saddles with pads (Equipe memory foam pad, Valdagno, Italy) if the rider subjectively felt a better fit was required between the saddle and the horse. Stirrup leather length on each side of the horse was measured prior to testing and corrected to equal length if required. During the test, riders (n = 4) showed tölt over one lap of the indoor oval track on both the left and the right reins (n = 2) for each of the two horses (n = 2). The testing was completed during four consecutive days, where the riding sessions were conducted between the hours of 16:00 and 17:00. In total, each riding session lasted approximately 40 minutes (20 mins per horse). Accordingly, both horses had at least 23 hours of rest prior to the following test session. The total procedure was performed on two separate occasions (n = 2) at least 3 weeks apart. The horses were ridden in a randomised order on both testing occasions. In total, 32 laps of tölt were recorded (4 riders*2 reins*2 horses*2 occasions).

**Fig 1:**
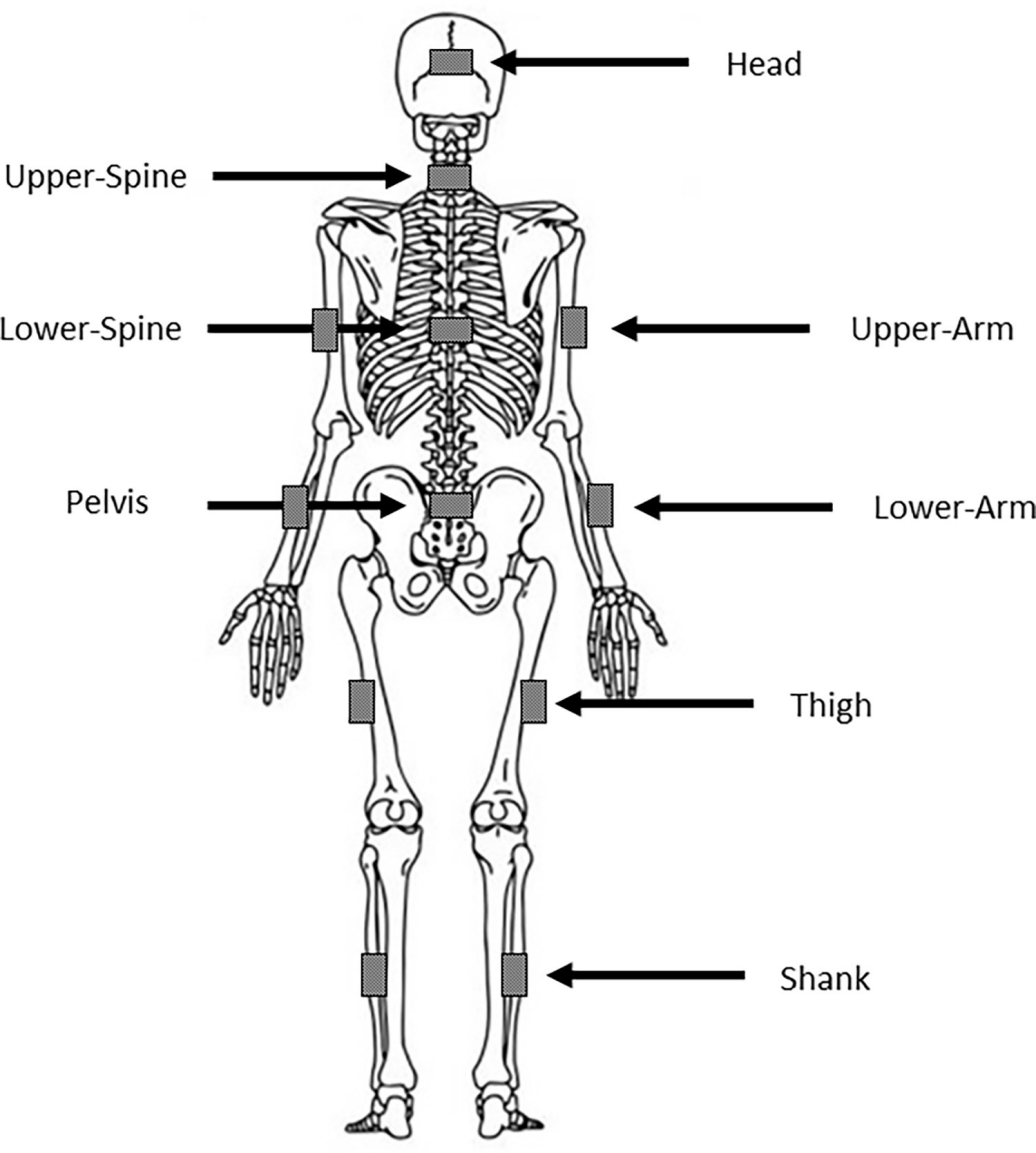
Positioning of the IMU sensors for the measurements. IMUs were positioned on each rider according to the manufacturer guidelines (https://www.noraxon.com/noraxon-download/myomotion-system-user-manual/).

Throughout all experimental tests riders were equipped with Pedar® foot pressure insoles (Novel electronics, Munich, Germany) and a Noraxon® 3D inertial motion analysis system (MyoMotion Research Pro, Noraxon Inc., Scottsdale, AZ, USA). The Noraxon® system comprises triaxial inertial measurement units (IMU; 100 Hz triaxial accelerometer, 100 triaxial Hz gyroscope, 100 Hz triaxial magnetometer) and has confirmed validity and reliability for measuring 3D angular movements [27, 28], which enabled the recording of kinematic data to assess rider asymmetry. Twelve IMUs were positioned by a trained exercise scientist onto anatomical locations over tight-fitting riding clothes prior to all riding sessions using manufacturer-provided elastic straps and bands. The IMUs were positioned on each rider according to the manufacturer guidelines, as illustrated in Fig 1 and further detailed at https://www.noraxon.com/noraxon-download/myomotion-system-user-manual/.

The Pedar® pressure insole data provided information at a sampling frequency of 50 Hz pertaining to the riders’ distribution of stirrup force between their left and right feet. The Pedar® pressure insole system has confirmed validity and reliability for the measurement of forces in field settings [29, 30].

The IMU and pressure insole systems were calibrated prior to all sessions according to the manufacturer’s guidelines using the manufacturer’s supplied software systems (IMU system: Noraxon® MyoMotion Research Pro, Version 3.16; Pressure insole system: Pedar /E (Expert) Software Version 8.23). Briefly, for the IMU system this involved a ‘zeroing’ of sensors whilst standing still with the arms by the sides. For the pressure insole system this involved the ‘zeroing’ of sensors when each foot was lifted off the ground.

All tests were filmed from the start to end using a Huawei Leica mobile phone (Huawei technologies Co, Shenzen, China) set at 60 Hz for evaluation of the horses’ stride parameters. Video recording took place from the centre of the riding hall. The indoor riding hall was separated into four sections for analyses, comprising the two straights and two curves. Seven stride cycles, from each of the four sections (two curves and two straights), were used and evaluated with Kinovea software (kinovea.org, version 0.8.15). The timestamp of each hoof contact was recorded for the subsequent analysis of LAP and DF, as described by Gunnarsson et al. [24]. LAP was calculated as a percentage of the time interval between every limb contact expressed as a proportion of the total duration, beginning with the right hind limb. DF was calculated as the duration of stance time relative to the front limb stride cycle, beginning with the right forelimb. In instances where hoof contact occurred between video frames the mean timestamp between frames was recorded. LAP and DF were calculated for all four sections of the riding hall in both left and right reins. Subsequently, the mean LAP and DF was calculated for both left and right reins. The same seven consecutive stride cycles were used to analyse both LAP and DF.

All measurement systems (i.e., Pedar, Noraxon and video) were synchronised at the precise moment when the rider mounted the horse. The moment when the foot was first positioned in the stirrup was determined from the IMU and video system and synchronised prior to being reconciled with the peak foot pressure at the moment of mounting.

### Measurements

The mean total absolute force of the left and right feet combined (F_Abs_) and the absolute mean difference between left and right absolute foot forces (F_diff_) for the riders were calculated and used for analyses. Elevation and depression of the left and right sides (roll) of the pelvis (RollP) and the thoracolumbar region (RollT), measured in degrees (°), were recorded continuously from the 3D inertial motion analysis system. The mean of all outcome variables over one complete lap (LAP, DF, F_Abs_, F_Diff_, RollP and RollT) were calculated from the riders’ two trials for both left and right reins with the same horse and used for analyses (i.e., each rider-horse interaction was considered as one independent measurement; n = 8). On six occasions out of 32 trials the Pedar® pressure insole data was lost due to system malfunction.

### Statistical analysis

Statistical analyses were performed using IBM SPSS Statistics (version 27.0; IBM Corporation, New York, USA) with the level of significance set at α ≤ 0.05. Repeated measures one-way ANOVA tests (within-subjects factor: riding rein direction) were used to assess the effect of rein direction (left *vs*. right) on rider asymmetry variables (F_Abs_, F_Diff_, RollP and RollT) and tölt performance (LAP, DF) on a group level (n = 8). For all ANOVA tests, effect sizes were calculated using partial eta-squared (ƞ^2^_*p*_). Within-subject Spearman rank correlation coefficients (*ρ*) were computed to determine the effect of rider asymmetry variables (F_Abs_, F_Diff_, RollP and RollT) on tölt performance (LAP and DF) on an individual level (n = 8). Strength of relationships were evaluated according to the following classifications for negative/positive *ρ* [31]: very small 0.0 to < 0.1; small 0.1 to < 0.3; moderate 0.3 to < 0.5; large 0.5 to < 0.7; very large 0.7 to < 0.9 and nearly perfect ≥ 0.9.

## Results

Table 1 displays the individual rider asymmetry and tölt performance variables for each rider-horse interaction. Table 2 displays the group means for rider asymmetry and tölt performance variables on the left and right reins. LAP was closer to 25% on the left rein compared to the right rein (mean difference: 1.8 ± 1.2%; F_(1,7)_ = 16.333; p = 0.005, η^2^_p_ = 0.700). In addition, DF was lower on the left rein compared to the right rein (mean difference: 1.9 ± 0.8%; F_(1,7)_ = 41.299; p < 0.001, η^2^_p_ = 0.855). Rein direction had no influence on F_Abs_ (F_(1,7)_ = 3.913; p = 0.088, η^2^_p_ = 0.359), F_Diff_ (F_(1,7)_ = 0.875; p = 0.381, η^2^_p_ = 0.111), RollP (F_(1,7)_ = 1.457; p = 0.267, η^2^_p_ = 0.172) or RollT (F_(1,7)_ = 2.286; p = 0.174, η^2^_p_ = 0.246).

**Table 1:** Tölt performance and rider asymmetry variables for each rider-horse interaction. F_Abs_ = Mean total absolute force of the left and right feet combined as measured from the foot force sensors; F_Diff_ = Absolute mean difference between the left and right foot forces as measured from the foot force sensors; RollP = Side- to- side movement (roll) of the pelvis; RollT = Side- to- side movement (roll) of the thoracolumbar region; LAP = Lateral advanced placement; DF = Duty factor; Left = Left rein; Right = Right rein.

Rider	Horse	F_Abs_ (N)		F_diff_ (N)		RollP (°)		RollT (°)		LAP (%)		DF (%)	
		Left	Right	Left	Right	Left	Right	Left	Right	Left	Right	Left	Right
1	1	99.2	100.0	19.3	12.1	2.5	2.9	1.1	1.2	23.0	22.0	37.9	39.1
1	2	63.0	70.9	18.4	19.8	2.5	2.6	0.9	0.9	23.5	21.5	41.2	43.1
2	1	73.6	69.0	19.3	6.0	1.7	2.3	1.7	1.3	22.0	21.0	40.5	42.2
2	2	58.6	68.9	13.6	20.0	2.3	2.5	1.0	1.2	24.0	21.5	42.5	44.5
3	1	48.6	45.7	6.3	3.8	3.2	3.2	1.0	1.0	22.5	22.0	36.2	39.5
3	2	34.8	44.0	6.9	8.9	3.3	2.8	1.0	0.8	23.5	21.0	41.6	44.3
4	1	27.4	30.9	11.3	36.8	2.5	2.6	2.8	1.7	22.0	21.5	40.8	42.0
4	2	6.4	14.1	1.8	26.3	2.4	2.7	3.7	3.4	25.0	21.0	44.7	45.6

**Table 2:** Mean ± SD rider asymmetry and performance variables during tölt riding on left and right reins. F_Abs_ = Mean total absolute force of the left and right feet combined as measured from the foot force sensors; F_Diff_ = Absolute mean difference between the left and right foot forces as measured from the foot force sensors; RollP = Side- to- side movement (roll) of the pelvis; RollT = Side- to- side movement (roll) of the thoracolumbar region; LAP = Lateral advanced placement DF = Duty factor.

	Left rein (n = 8)	Right rein (n = 8)
**F**_**Abs**_ **(N)**	51 ± 29	55 ± 27
**F**_**diff**_ **(N)**	12 ± 7	17 ± 11
**RollP (°)**	2.6 ± 0.5	2.7 ± 0.3
**RollT (°)**	1.6 ± 1.0	1.4 ± 0.9
**LAP (%)**	23.2 ± 1.0*	21.4 ± 0.4
**DF (%)**	40.7 ± 2.6*	42.5 ± 2.3

* Significantly different from right rein, p < 0.05.

Spearman correlation coefficients between RollT/RollP and LAP/DF were highly variable (Table 3). Individual relationships between RollT and LAP ranged from small negative to very large positive and reached significance for one rider with wide confidence intervals (*ρ =* 0.730; 95% confidence interval: 0.027 to 0.950; p = 0.040). Individual relationships between RollP and DF ranged from very large negative to very large positive and reached significance for two riders with wide confidence intervals (*ρ =* 0.731; 95% confidence interval: 0.027 to 0.950; p = 0.040; *ρ = -*0.723; 95% confidence interval: -0.948 to -0.011; p = 0.043). No other individual relationships reached significance.

**Table 3:** Individual Spearman correlation coefficients between rider asymmetry variables and tölt performance. F_Abs_ = Mean total absolute force of the left and right feet combined as measured from the foot force sensors; F_Diff_ = Absolute mean difference between the left and right foot forces as measured from the foot force sensors; RollP = Side- to- side movement (roll) of the pelvis; RollT = Side- to- side movement (roll) of the thoracolumbar region; LAP = Lateral advanced placement DF = Duty factor.

Rider	RollT *vs*. LAP	RollP *vs*. LAP	RollT *vs*. DF	RollP *vs*. DF
1	0.172	-0.147	-0.488	-0.331
2	-0.106	0.148	-0.349	0.731*
3	0.730*	0.501	-0.563	-0.119
4	0.691	-0.152	-0.060	-0.723*

* *Significantly correlated*, *p* < 0.05.

## Discussion

This pilot study investigated biomechanical variables in Icelandic horse riders during tölt, with a main aim to identify whether rider asymmetry influences tölt performance. The findings showed that: 1) tölt performance was better on the left rein compared to the right rein, reflected by better LAP and DF scores; 2) group level asymmetry variables were not different between left and right reins; 3) individual relationships between rider asymmetry variables and tölt performance were highly variable but reached significance in some instances, suggesting that rider asymmetry might influence tölt performance.

The high degree of individuality in rider asymmetry in this pilot study might indicate that some riders were unable to stabilize their axial body segments from the pelvis upwards. Previous research has demonstrated that very few riders can control their pelvis without compensatory movements [17]. As such, this asymmetry and compensatory movement, might influence horses’ locomotion [11]. One compensatory movement to help stabilize the body when riding, might be the use of the stirrups to counterbalance the asymmetry [12]. This might lead to lateral imbalances in stirrup pressure, however, in this pilot study there were no associations between stirrup force asymmetries and tölt performance. Nevertheless, future research should consider investigating parameters related to stirrup imbalances, in addition with trunk and pelvic imbalances, and their relationships with tölt performance.

Very few studies have investigated tölt previously, so it is unclear why tölt performance was significantly better on the left rein compared to the right rein in this investigation. Whilst speculative, one explanation could relate to horse laterality. In support of this hypothesis, many research studies of human athletes have shown different performance levels between left and right directions during curve sprinting [e.g., 32–34]. As such, it is possible that horses also perform better in one direction over the other. Accordingly, it might be important for researchers and coaches to consider that tölt performance could be affected by rein directions. Another possible explanation for the differences in tölt performance between left and right directions is subclinical lameness. No objective gait analyses were performed on the horses in this pilot study. Although no lameness was indicated and the veterinarian cleared the horses for competition on all occasions, this does not necessarily preclude lameness when ridden [35]. Another factor to consider is that saddle fit was performed by each rider using their own equipment, rather than by a specific expert or with a standardised saddle. As such, saddle fit might have affected both horse and rider locomotion [10, 26]. Furthermore, rider morphology could potentially influence gait performance [25, 35, 36]. However, the body height and mass of the riders relative to the horse were not measured in this pilot study.

The large variability in the strength of the Spearman correlation coefficients for individual riders demonstrates that the relationships between rider asymmetry and tölt performance are highly individualised. The correlation between RollT and LAP reached significance for one rider, albeit with wide confidence intervals. Additionally, the correlation between RollP and DF reached significance for two riders, with one rider displaying a strong positive relationship between RollP and DF and the other displaying a strong negative relationship. The inter-individual variability in the strength of correlations makes it challenging to provide general recommendations at a group level. In some individuals more movement in the thoracolumbar or pelvic regions were related to better performance (riders 3 and 4, respectively). However, for another individual (rider 2) less pelvic movement was related to better tölt performance. This might reflect the individual nature of the interaction between the rider and the horse. Regardless, monitoring rider asymmetry variables during tölt could be useful for equestrians given that rider asymmetry appears to influence performance for some riders. However, further research is required to assess the robustness of intra-individual relationships between these parameters.

This pilot study has several limitations that must be considered. Firstly, the statistical power is low due to the small number of participants (n = 4) and horses (n = 2) and the results should therefore be treated accordingly. The repeatability of the IMU marker placement was not assessed, which could affect the results. The sample frequency of the video capture used to determine LAP and DF (i.e., 60 Hz) was in the lower range of what might be expected for biomechanical measurements. Additionally, LAP was calculated beginning with the right hind limb for both reins. This might have affected LAP because the temporal patterns of footfall might differ between left and right sides.

This pilot study highlights that riding rein direction can influence tölt performance. However, no rider asymmetry variables were different between left and right reins at the group level. Relationships between rider asymmetry and tölt performance at the individual level were highly variable and reached significance in some instances, indicating that the relationship between rider asymmetry and tölt performance is highly individual. This type of biomechanical data has the potential to provide important feedback to guide equestrians and coaches in the quest for improved competitive performance.

## Supporting information

S1 File (ZIP)

S1 Data (CSV)

S2 Data (CSV)

S3 Data (CSV)
